# An Internal Ribosome Entry Site (IRES) Mutant Library for Tuning Expression Level of Multiple Genes in Mammalian Cells

**DOI:** 10.1371/journal.pone.0082100

**Published:** 2013-12-09

**Authors:** Esther Y. C. Koh, Steven C. L. Ho, Mariati, Zhiwei Song, Xuezhi Bi, Muriel Bardor, Yuansheng Yang

**Affiliations:** 1 Bioprocessing Technology Institute, Agency for Science, Technology and Research, Singapore, Singapore; 2 Laboratoire Glyco-MEV (Glycobiologie et Matrice extracellulaire végétale), Université de Rouen, Mont-Saint-Aignan, France; 3 School of Chemical and Biomedical Engineering, Nanyang Technological University, Singapore, Singapore; St. Jude Children's Hospital, United States of America

## Abstract

A set of mutated Encephalomyocarditis virus (EMCV) internal ribosome entry site (IRES) elements with varying strengths is generated by mutating the translation initiation codons of 10^th^, 11^th^, and 12^th^ AUG to non-AUG triplets. They are able to control the relative expression of multiple genes over a wide range in mammalian cells in both transient and stable transfections. The relative strength of each IRES mutant remains similar in different mammalian cell lines and is not gene specific. The expressed proteins have correct molecular weights. Optimization of light chain over heavy chain expression by these IRES mutants enhances monoclonal antibody expression level and quality in stable transfections. Uses of this set of IRES mutants can be extended to other applications such as synthetic biology, investigating interactions between proteins and its complexes, cell engineering, multi-subunit protein production, gene therapy, and reprogramming of somatic cells into stem cells.

## Introduction

Simultaneous expression of multiple genes in mammalian cells at finely controlled amounts or ratios is required for applications such as synthetic biology, investigating interactions between proteins and its complexes, cell engineering, multi-subunit protein production, gene therapy, and reprogramming of somatic cells into stem cells [1], [2], [3], [4]. Three common strategies for controlling multiple gene expression in mammalian cells are (i) co-transfection of multiple vectors at different relative amounts [5], [6], [7], (ii) single vector containing multiple promoters or polyadenylation signals with different strengths [8], [9], [10], and (iii) insertion of splicing signals with varied splicing efficiencies between genes [11]. Co-transfection is an inaccurate approach as the relative amount of different genes incorporated varies from cell-to-cell due to variations in transfection efficiency [12], [13]. Using a single vector with multiple promoters or polyadenylation signals ensures the introduction of several genes into each cell at identical amounts and provides accurate control of gene expression in transient transfections [9]. However, the expression ratio still varies between cells in a stably transfected cell pool [7], [13], [14] as the arrangement of multiple promoters in close proximity causes transcriptional interference, where the active expression of one gene suppresses expression of the other genes. The degree to which gene expression is suppressed depends on the integration site in the genome [15]. The use of splicing signals allows stricter control of relative gene expression as all genes are expressed in one transcript [11]. This method requires elimination of cryptic splicing sites in protein coding sequences to prevent incorrect RNA splicing.

Co-expression of multiple genes in one mRNA for strict control of relative gene expression can also be achieved by using either 2A elements or internal ribosome entry site (IRES). 2A linked genes are expressed in one single open reading frame (ORF) and “self-cleavage” occurs co-translationally to give equal amounts of co-expressed proteins [16]. This method does not allow modulation of the expression ratio between the proteins of interest. Moreover, incomplete cleavage of 2A peptides often results in attachment of additional unwanted amino acids residues to proteins and formation of fusion proteins [17], [18], [19], [20]. When IRES elements are included between multiple open reading frames (ORFs), the first ORF is translated by the canonical cap-dependent mechanism while the rest are translated through a cap-independent mechanism [21], [22], [23]. It has already been shown that IRES allows strict control of the relative gene expression in both transient and stable transfections [13], [24], [25]. In contrast to the 2A element, products generated using IRES do not form any undesirable modified or fusion proteins [17], [20]. More importantly, as genes are translated independently, the relative expression of different genes can be adjusted by varying the strength of the IRES applied to each gene. Although expression can be altered by using different naturally available IRES, the range of expression levels obtained is narrow due to the limited range of IRES elements [24]. The range of IRES activity can be widened by mutating its sequence [26], [27]. An earlier study generated a set of eleven IRES mutants by error prone PCR which enabled controlled gene expression level across a 20-fold range [28]. However, the strengths of these IRES mutants appear to be cell specific as four IRES mutants that exhibited up to 36% strength differences in HEK293T cells showed no apparent difference in CHO K1 cells.

Encephalomyocarditis virus (EMCV) IRES is the most widely used IRES element for multiple gene expression in mammalian cells because of its superior activity in different cell lines and ability to mediate accurate translation [26], [29], [30]. The region that contributes to efficient EMCV IRES translation contains twelve AUGs [31]. Translation initiation occurs primarily at the 11^th^ AUG (AUG-11), partially at the 12^th^ AUG (AUG-12), and almost none at the 10^th^ AUG (AUG-10) [32], [33]. In cap-dependent translation, ribosomes can recognize codons other than AUG and initiate translation with reduced efficiency and gene expression. These non-AUG codons in mammalian cells have a relative rank order of translation efficiency of AUG>CUG>GUG>ACG>AUA>AUU>UUG [34], [35], [36]. If IRES-mediated cap-independent translation follows the same principle, generation of EMCV IRES mutants with AUG-10, AUG-11, and AUG-12 mutated to non-AUG triplets should provide a new tool for accurate control of the expression level of multiple genes in mammalian cells.

In this work, we generated a series of mutated EMCV IRES by either deletion or mutation of AUG-10, AUG-11, and AUG-12 in the wild type EMCV IRES (IRESwt). They were evaluated in different mammalian cell lines and their use was demonstrated for optimization of light chain (LC) to heavy chain (HC) expression ratios for enhancing monoclonal antibody (mAb) expression level and quality. Different ratios of LC over HC expression affect both mAb expression levels and quality [7], [14], [37]. The optimum LC:HC ratio for mAb production depends on the cell type used for expression, whether production is performed using transient or stable transfections, and possibly the mAb molecule itself [7], [28]. Here, we demonstrated that using different IRES mutants to express LC and HC allowed the control of LC:HC ratios at a level most beneficial for mAb expression and quality. This set of IRES mutants can be also used in other applications such as synthetic biology, investigating interactions between proteins and its complexes, cell engineering, multi-subunit protein production, gene therapy, and reprogramming of somatic cells into stem cells. The crippled EMCV IRES mutants with reduced translation efficiencies would be particularly useful for expressing proteins at more biologically relevant levels in the cell, or for expressing proteins that are toxic to the cells if over expressed.

## Materials and Methods

### Cell Culture and Media

Adherent CHO K1, HEK293, BHK, 3T3, and COS7 cells (ATCC, Manassas, VA) were grown in the Dulbecco's modified Eagle's medium (DMEM) + GlutaMax™ (Life Technologies, Carlsbad, CA) supplemented with 10% fetal bovine serum (Sigma, St. Louis, MO) in a static humidified incubator with 5% CO_2_ at 37°C. Suspension dihydrofolate reductase (DHFR)-deficient CHO DG44 cells (Life Technologies) were grown in a protein-free medium consisting of 50% HyQ PF (HyClone, Logan, UT) and 50% CD CHO (Life Technologies) supplemented with 1 g/L sodium carbonate (Sigma), 6 mM glutamine (Sigma), 0.1% Pluronic F-68 (Life Technologies), and 1% hypoxanthine and thymine (HT) (Life Technologies) in humidified Kuhner shakers (Adolf Kühner AG, Birsfelden, Switzerland) with 8% CO_2_ at 37°C.

### Generation of EMCV IRES Variants

The IRESwt with sequence corresponding to the region from 260 to 848 in the EMCV-R genome (Genbank: M81861) was cloned from the pIRES2-DsRed vector (Clontech Laboratories, CA). It contained two mutations compared to the sequence deposited in the Genbank (G at 739 to A and insertion of one A after 769) and is less active than the wild-type version [26]. The mutated EMCV IRES variants were generated by either mutation of AUG-10, AUG-11, and AUG-12 individually or in combination to GUG, CUG, ACG, AUA, UUG, or deletion of AUG-11 and AUG-12 and surrounding sequence, or both (Table I). The mutations were obtained by using synthetic primers containing specified mutations during PCR amplification.

**Table I:** Relative strength of IRES variants in expressing a gene.

EMCV IRES 3′-end Sequence	Strength (%)			Variant ID
10^th^ 11^th^ 12^th^				
AUGAUAAUAUGGCCACAACCAUG (WT)	100.00	±	0.00	IRESwt
GUGAUAAUAUGGCCACAACCAUG	90.48	±	0.12	IRESv1
AUGAUAAUAUGGCCACAACCGUG	83.59	±	1.72	IRESv3
AUGAUAAUGUGGCCACAACCAUG	35.48	±	3.50	IRESv7
GUGAUAAUAUGGCCACAACCGUG	67.42	±	2.91	IRESv4
GUGAUAAUGUGGCCACAACCAUG	29.45	±	1.86	IRESv10
AUGAUAAUGUGGCCACAACCGUG	0.98	±	0.02	IRESv19
GUGAUAAUGUGGCCACAACCGUG	1.37	±	0.02	IRESv18
AUGAUAAUCUGGCCACAACCAUG	45.18	±	2.12	IRESv5
AUGAUAAUAUAGCCACAACCAUG	39.91	±	0.80	IRESv6
AUGAUAAUUUGGCCACAACCAUG	34.39	±	0.72	IRESv8
AUGAUAAUACGGCCACAACCAUG	33.25	±	0.36	IRESv9
AUGAUAAUCUGGCCACAACCCUG	9.47	±	1.90	IRESv13
CUGAUAAUCUGGCCACAACCCUG	13.58	±	1.50	IRESv12
AUGAUAAUACGGCCACAACCACG	4.02	±	0.87	IRESv14
ACGAUAAUACGGCCACAACCACG	3.23	±	0.18	IRESv15
AUGAUAAUAUAGCCACAACCAUA	1.79	±	0.09	IRESv16
AUAAUAAUAUAGCCACAACCAUA	1.67	±	0.16	IRESv17
AUGAUAAUUUGGCCACAACCUUG	0.58	±	0.09	IRESv21
UUGAUAAUUUGGCCACAACCUUG	0.81	±	0.03	IRESv20
AUGAUAAUAUG	86.42	±	0.03	IRESv2
AUG	24.35	±	4.04	IRESv11
AUGAUAAUGUG	0.34	±	0.20	IRESv24
GUGAUAAUGUG	0.46	±	0.10	IRESv23
GUG	0.57	±	0.12	IRESv22

### Vector Construction

**Figure 1:**
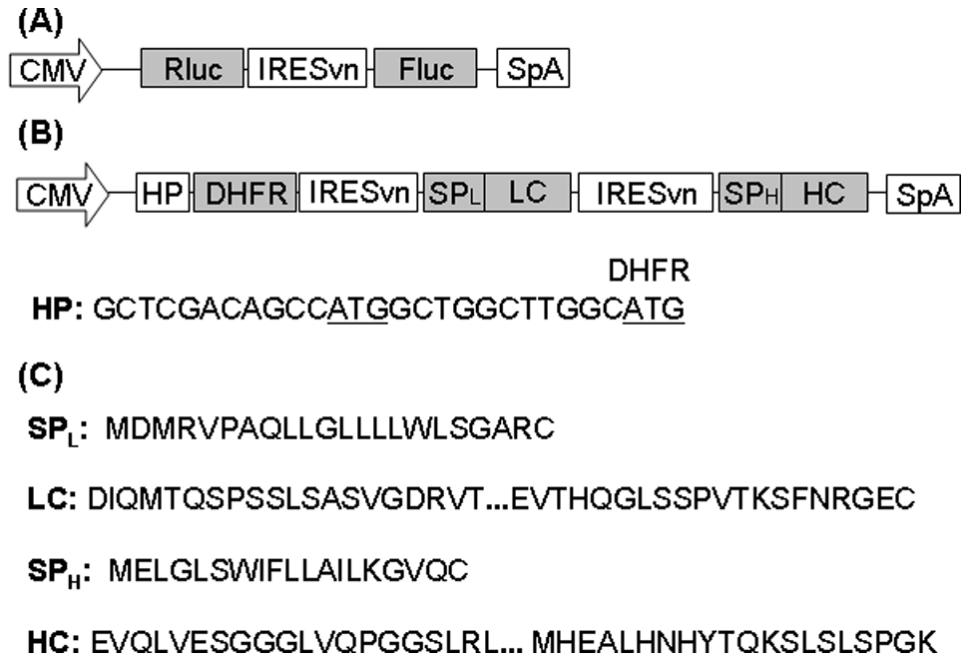
Schematic representation of vectors. (A) Structure of dual-luciferase bicistronic vectors for determination of IRES variants' strengths. (B) Structure of monoclonal antibody expressing tricistronic vectors with specific IRES variants applied on the light chain (LC) or heavy chain (HC) genes. (C) Amino acid sequences of signal peptide, N- and C-terminal end of LC and HC. CMV, human cytomegalovirus IE gene promoter; IRESwt, wild type encephalomyocarditis virus (EMCV) internal ribosome entry site (IRES); IRESvn, a specific EMCV IRES variant; SpA, SV40 polyadenylation signal; Rluc, renilla luciferase coding region; Fluc, firefly luciferase coding region; SP_L_, light chain signal peptide; LC, light chain coding region; SP_H_, heavy chain signal peptide; HC, heavy chain coding region; DHFR, dihydrofolate reductase coding region; HP, DNA sequence that can form a hairpin structure and contains an upstream AUG that is out of frame with the coding sequence.

The IRES-mediated dual-luciferase bicistronic vectors containing different IRES variants on the firefly luciferase (Fluc) gene were constructed by replacing the BGHpA-CMV region in a previously described vector [10] with specific IRES variants (Figure 1A). The IRES-mediated tricistronic vectors containing different IRES variants on LC and HC were constructed by replacing the IRESwt-LC and IRESwt-HC regions in a previously described tricistronic vector expressing anti-HER2 mAb [37] with IRESvn-LC and IRESvn-HC, respectively (Figure 1B). IRESvn-LC and IRESvn-HC were synthesized by overlapping PCR. A short DNA sequence that can form a hairpin structure (HP) and contains an upstream AUG that is out of frame with the coding sequence of DHFR was inserted 101 base pair downstream of the predicted human cytomegalovirus IE gene promoter (CMV) transcription start site (Figure 1B). The out-of-frame AUG is present in an optimal sequence context for efficient initiation of translation. Insertion of such a DNA sequence immediately in front of the DHFR start codon is expected to reduce DHFR expression by about 50-fold [38] and enhance the stringency of selection for high producers in stable transfections [13].

### Transient Transfection

Transient transfections for evaluating the strength of IRES variants in different mammalian cell lines were carried out in 6-well tissue culture plate (NUNC™, Roskilde, Denmark) using Fugene 6 (Roche, Indianapolis, IN). 24 h prior to transfection, 2 mL of exponentially growing cells at a cell density of 3×10^5^ cells/mL were seeded into each well of the 6-well plates. Transfection of cultures in each well with the appropriate dual-luciferase vector was done in duplicates using a recipe of 6 µL of Fugene 6:2 µg of DNA. At 48 h post-transfection, cells were detached with trypsin and analyzed for renilla luciferase (Rluc) and Fluc activities using Dual-Glo Luciferase Assay system (Promega, Madison, WI) in white opaque 96-well assay plates (Corning) in quadruplicates. The transfections and luciferase assays were repeated one more time using independently prepared plasmids and cultures.

### Generation of Stably Transfected mAb Producing Cell Lines

Suspension CHO DG44 cells were transfected with the tricistronic vectors containing different IRES variants on the LC and HC genes (Figure 1B) by using electroporation on a Nucleofector (Lonza, Cologne, Germany). In each transfection, 1×10^7^ cells were transfected with 5 µg of linearized plasmids. The transfected cells were then resuspended in 2 mL of protein-free medium preloaded in 6-well suspension culture plates (NUNC™). At 24 h post-transfection, they were collected by centrifuge at 1000 rpm for 5 minutes, and then resuspended in 15 mL of protein-free medium without HT and then followed by gene amplification in protein-free medium containing stepwise increased concentrations of methotrexate (MTX) at 50 nM and 250 nM respectively. To determine the productivity of mAb in stable transfection pools, 25 mL of cultures at a cell density of 2×10^5^ cells/mL were seeded into 125 mL shake flask. Cell density and viability were monitored using Cedex counter (Innovatis, Bielefeld, Germany) until viability dropped below 50%. Supernatant was collected at the end of culture and analyzed for mAb concentration using a nephelometric method on an IMMAGE 800 immunochemistry system (Beckman Coulter, Buckinghamshire, England).

### ELISA Analysis of Intracellular Polypeptides of LC:HC Ratios

The intracellular polypeptides of LC:HC ratios in stable transfection pools generated using tricistronic vectors containing different IRES variants on LC and HC were determined using ELISA as previously described [13]. 1×10^7^ cells were collected from 125 mL shake flask batch cultures growing at exponential phase. They were lysed in RIPA buffer (Thermo Scientific, Waltham, MA) supplemented with ProteoBlock protease inhibitor cocktail (Fermentas, Thermo Scientific). The cell lysates were centrifuged at ∼1800×g for 30 min at 4°C. The supernatants were then collected and quantified for concentrations of LC and HC polypeptides using alkaline phosphatase-conjugated goat anti-human IgG (Fc specific) for HC detection and goat anti-human IgG (LC specific) for LC detection, respectively. Both detection antibodies were purchased from Sigma-Aldrich. The intracellular LC:HC polypeptide ratio in each stable transfection pool was determined as the measured LC concentration divided by the HC concentration.

### Western Blotting Analyses of Cell lysates and Supernatant

Western blotting analyses of the cell lysates and supernatant were carried out as described previously [37]. 10 µg of total proteins were loaded into each lane of NuPAGE 4–12% Bis-Tris gels (Life Technologies) for analysis of LC and HC polypeptides in the cell lysates. For analysis of supernatant, the sample loaded into each lane contained 1 ng of mAb as determined by ELISA using Fc-specific detection antibodies. 10 pg of product was loaded for the sample with LC:HC of 21.24 to prevent overexposure due to the high levels of accumulated LC_2_ dimers and LC monomers. The LC and HC polypeptides were detected simultaneously using two antibodies, HRP conjugated goat anti-human IgG Fc antibody and HRP conjugated goat anti-human IgG Kappa LC antibody. Both detection antibodies were purchased from Bethyl Laboratories (Montgomery, TX).

### mAb Purification and Aggregation analysis

mAb in the supernatant was purified using protein A column on a GE AKTA explorer 100 (GE Healthcare, Uppsala, Sweden). The aggregation of protein A purified mAb was determined using size exclusion chromatography (SEC) coupled to a UV-visible detector and a dynamic light scattering detector. The instruments setup, chromatography conditions, and data analysis were as previously described (Ho et al. 2012).

### LC-MS/MS Analysis of Signal Peptide Cleavage

Protein A purified mAb in stable transfection pools at different LC:HC ratios were analyzed for the signal peptide cleavage sites using NanoLC-MS/MS as described previously [17].

## Results

### Strengths of EMCV IRES Variants in CHO K1 Cells

**Figure 2:**
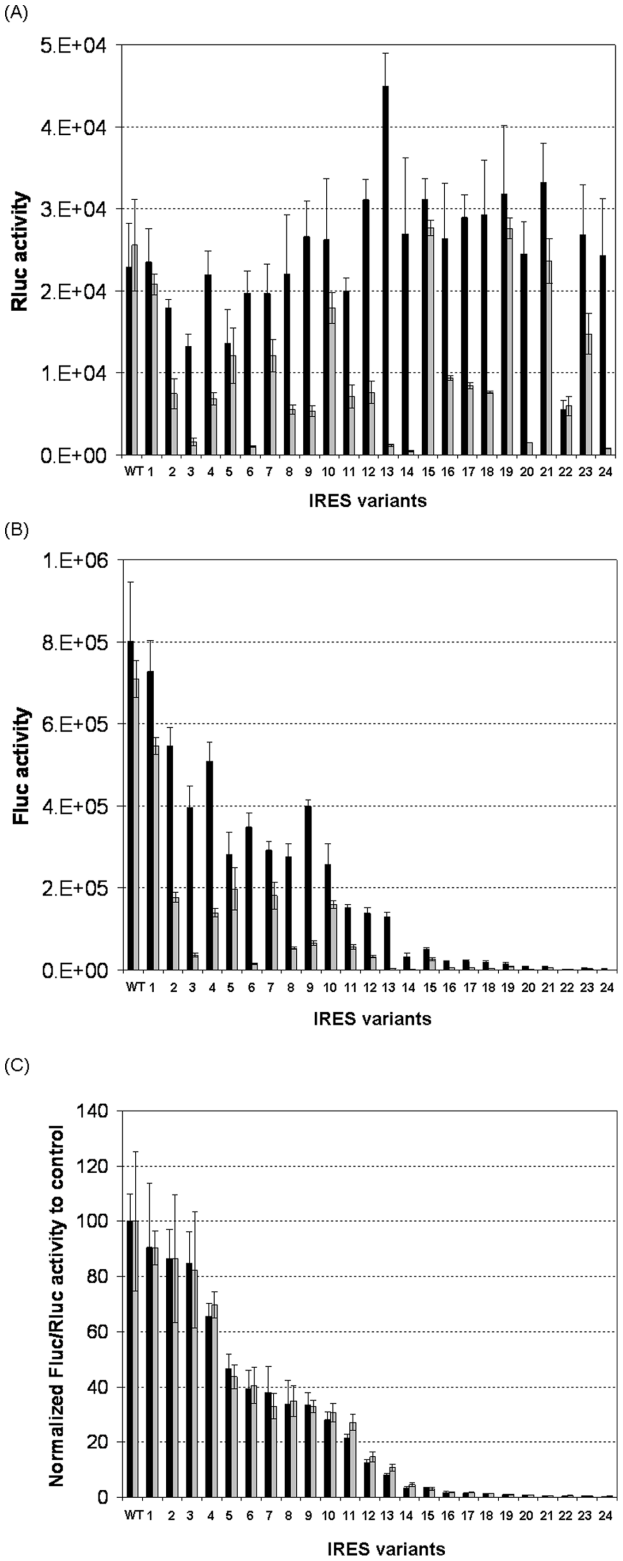
Relative strength of EMCV IRES variants in CHO K1 cells in transient transfections. Equal amounts of dual-luciferase bicistronic vectors (refer Figure 1A) encoding renilla luciferase (Rluc) and firefly luciferase (Fluc) genes were transfected into CHO K1 cells. At 48 h post-transfection, cell pellets were collected for analysis of Fluc and Rluc luciferase activities by using Dual-Glo Luciferase Assay system. Transfection of each vector was done in duplicates and repeated a second time using independently prepared plasmids and cultures. (A) and (B) Measured Fluc and Rluc activities. (C) Ratios of luciferase activities of Fluc to Rluc gene for each IRES variant normalized to the wild-type IRES (WT). Each individual bar represents the average and standard deviation of eight measurements from two transfections in one experiment. Black bars represent the results from experiment 1 and gray bars from experiment 2.

The strengths of IRES variants were determined using dual-luciferase bicistronic vectors (Figure 1A) in transient transfections. The constructs were generated such that the 12^th^ start codon for the EMCV IRES was used as the Fluc start codon. It has been reported that the expression of the first cistron (cap-dependent translation) is not affected by the downstream IRES-driven cistron (cap-independent translation) [39], [40]. Thus Rluc was used as an internal standard to normalize the transfection efficiency for accurate determination of Fluc expression to reflect the strength of IRES variants. However, we observed that both Rluc and Fluc readings varied drastically both across different IRES variants and between the two independent experiments (Figure 2A and 2B). Rluc reading variations should be due to variations in transfection efficiency and Fluc reading variations could be due to both varied transfection efficiency and different strengths of IRES variants. After normalization of the Fluc reading to both the Rluc reading and the IRESwt, the effect different IRES variants had on Fluc expression were clear and highly reproducible in the two experiments (Figure 2C). The average relative strength and standard deviation of each IRES variant determined from the two independent experiments were calculated and reported in Table I. These 24 IRES variants enabled expression of Fluc to be controlled over a 300-fold range with small intervals and were sorted according to their relative strengths in descending order with IRESv1 referring to the strongest variant and IRESv24 referring to the weakest variant (Figure 2 and Table I).

All IRES variants exhibited reduced Fluc expression as compared to the IRESwt (Table I). The magnitude of reduction varied depending on the position and the number of mutated AUGs. Mutation of AUG-10 and AUG-12 to GUG reduced IRES strength to 90.48% and 83.59%, respectively, while mutation of AUG-11 reduced the strength more significantly to 35.48%. These results are consistent with previous reports that translation by IRESwt is initiated primarily at AUG-11, partially at AUG-12, and very rarely at AUG-10 [32], [33], [41]. Mutation of two AUGs had combinatorial effect in reducing the strength of IRES. For instance, mutation of AUG-10 and AUG-12 alone to GUG reduced the strength to 90.48% and 83.59%, respectively, while mutation of both to GUG reduced the strength to 67.42%. Similarly, mutation of both AUG-10 and AUG-11 reduced the strength to 29.45% and mutation of both AUG-11 and AUG-12 reduced the strength to 0.98%. Interestingly, mutation of all three AUGs to GUG reduced the strength to 1.37%, showing no significant difference compared to mutation of AUG-11 and AUG-12 together as analyzed by two-sample *t*-test.

The type of bases used for AUG mutations affected the strength of IRES differently. For example, mutation of AUG-11 to CUG was least effective in reducing strength, giving strength of 45.18%, followed by AUA (39.91%), GUG (35.48%), UUG (34.39%), and ACG (33.25%). Two-sample *t*-test indicated that the differences between CUG and GUG (p<0.1), CUG and UUG (*p*<0.1), CUG and ACG (*p*<0.1), AUA and UUG (*p*<0.1), AUA and ACG (*p*<0.1) were statistically significant. The effect that the type of base had on IRES strength changed when both AUG-11 and AUG-12 or all three AUGs were mutated, giving an order of CUG>ACG>AUA>GUG>UUG with strengths covering a range from 13.58% to 0.58%. Consistent with the observation for GUG, mutating all three AUGs to CUG, AUA, UUG, or ACG did not exhibit significantly reduced strength compared to mutations of AUG-11 and AUG-12 to the corresponding triplets as analyzed by using two-sample *t*-test. Deleting either AUG-12 alone or both AUG-12 and AUG-11 together with surrounding sequences was also effective in reducing IRES strength, resulting in strengths of 86.42% and 24.35%, respectively. Further mutations combined with the deletions generated three IRES variants IRESv24, IRESv23, and IRESv22 with strengths reduced to 0.34%, 0.46%, and 0.57%.

### Evaluation of EMCV IRES Variants in Different Mammalian Cell Lines

**Figure 3:**
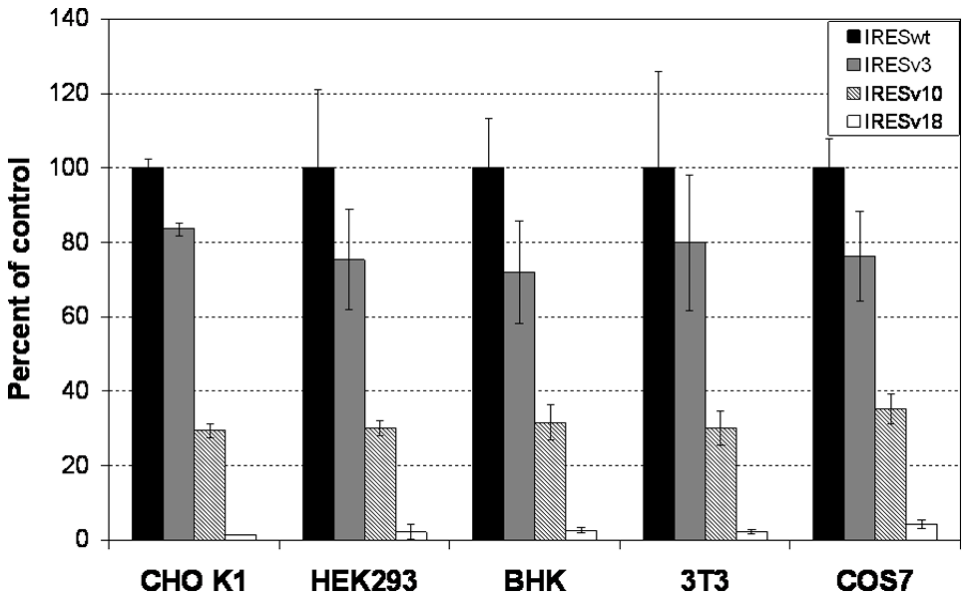
Relative strength of EMCV IRES variants in different mammalian cell lines in transient transfections. The strengths of IRES variants in different cell lines were obtained by transfection of dual-luciferase vectors (refer figure 1A) containing different IRES variants including IRESv3, IRESv10, or IRESv18 on the Fluc gene. At 48 h post-transfection, the luciferase activities of Rluc and Fluc gene were quantified by Dual-Glo Luciferase Assay Systems. Results represent the strength of each IRES variant calculated as the ratios of luciferase activities of Fluc to Rluc normalized to the control, the wild type EMCV IRES (IRESwt). Each value represents the average and standard deviation of sixteen measurements from four independent transfections for CHO K1 cells and eight measurements from two independent transfections for other cell lines.

To evaluate the application of IRES variants in different mammalian cell lines, IRESwt and three representative IRES variants, IRESv3, IRESv10, and IRESv18 that exhibited significantly different strengths in CHO K1 cells were tested in another four cell lines. The five cell lines were chosen from a range of species and tissues: CHO K1 from Chinese hamster ovary, HEK293 from human embryonic kidney, BHK from baby hamster kidney, 3T3 from mouse embryo, and COS7 from African Green Monkey kidney. The first three cell lines have been used widely for production of recombinant proteins in industry and the last two have been used in fundamental biological studies. The three IRES variants exhibited similar relative strengths to the IRESwt in expressing a gene in all different cell lines. The IRESwt was the strongest in all cases, followed by IRESv3, IRESv10, and IRESv18 (Figure 3).

### Application of EMCV IRES Variants for Controlling LC over HC Expressions

**Figure 4:**
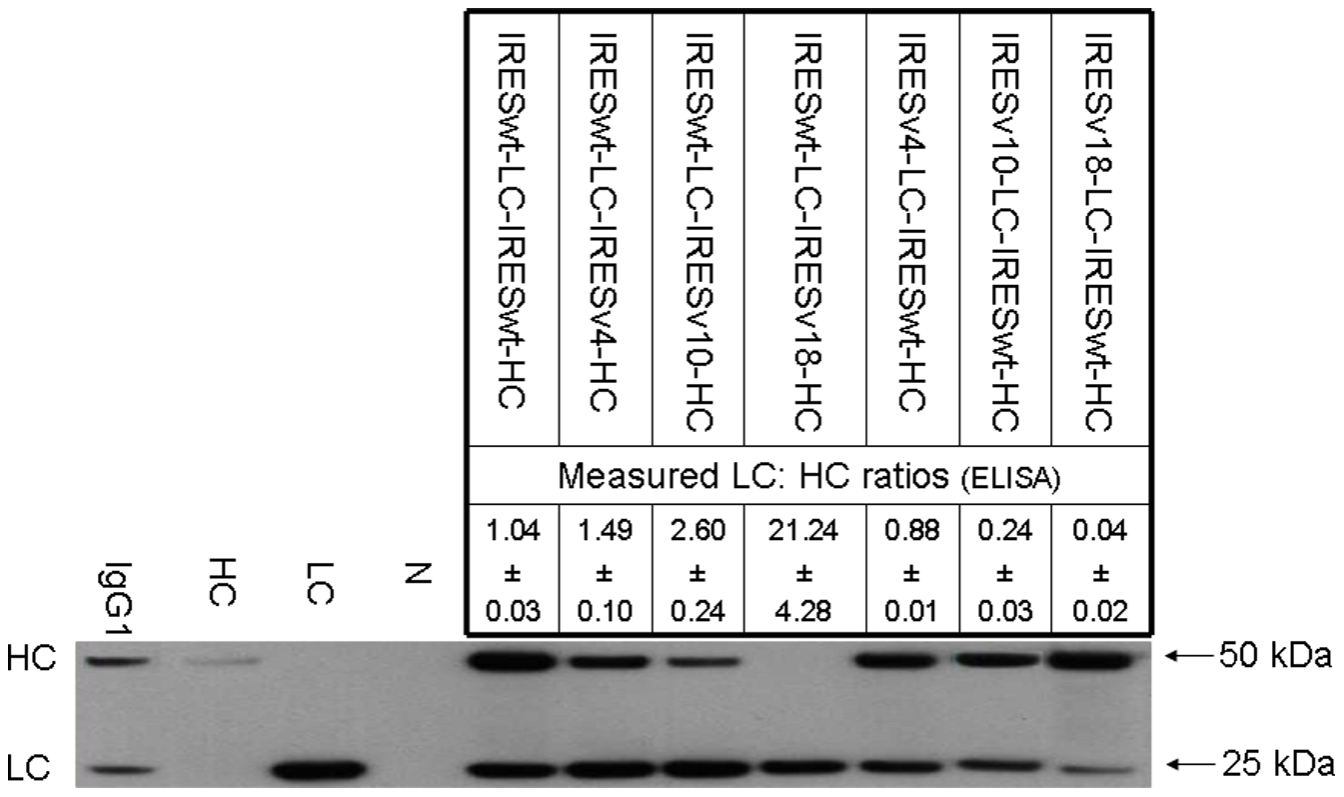
Control of LC and HC expression using different IRES variants in stable transfections. CHO DG44 stable transfection pools were generated by transfection of tricistronic vectors with different IRES variants applied on the LC and HC cDNA (refer to Figure 1B). LC:HC ratios listed in the table were calculated as the concentration of intracellular LC polypeptides determined by ELISA divided by the HC polypeptides. Each value represents the average and standard deviation of four measurements from two independent stable transfection pools. The intracellular abundance of LC and HC polypeptides were also analyzed using western blot under reducing conditions. Cell lysates containing equal amounts of proteins were loaded into each lane. A commercial human affinity purified myeloma Ig1 (Sigma-Aldrich) and supernatants from cells transfected with either a vector expressing only HC or a vector expressing only LC were used as positive control, and supernatant from non-transfected cells as negative control (N).

The application of EMCV IRES variants for controlling the expression level of multiple genes in stable transfections was demonstrated by expressing a multi-subunit protein, IgG mAb. An IgG molecule is composed of two identical LC and two identical HC polypeptides linked by disulfide bonds. We first constructed a control anti-HER2 mAb expressing tricistronic vector with both LC and HC under the control of IRESwt (Figure 1B). Another six vectors were then constructed by replacing IRESwt with IRES variants of IRESv4, IRESv10, and IRESv18 for different LC:HC ratios. These vectors were transfected into CHO DG44 cells to generate stably transfected pools. Intracellular LC:HC polypeptide ratios in each stable pool were determined using ELISA (Figure 4). The control vector with IRESwt applied on both LC and HC presented a LC:HC ratio of 1.04. Application of weaker IRESv4, IRESv10, and IRESv18 on HC increased the LC:HC ratios to 1.49, 2.60, and 21.24, respectively. When the same set of IRES variants were applied on LC, they decreased the LC:HC ratios to 0.88, 0.24, and 0.04, respectively. Western blotting analysis of the same intracellular lysates prepared for ELISA was performed to verify the LC:HC ratios. Cellular lysates containing equal amount of total proteins were reduced and loaded into each lane of NuPAGE gel. The band intensities for LC and HC under the control of IRESwt were not significantly changed across different vectors, indicating that the expression of LC and HC can be independently altered by IRES variants without interference by each other. The band intensities corresponding to LC and HC polypeptide abundance were steadily decreased by weak IRES variants, suggesting that the altered LC:HC ratios were achieved by reducing either LC or HC expressions. The band corresponding to HC under the control of IRESv18 was not visible due to low expression.

**Figure 5:**
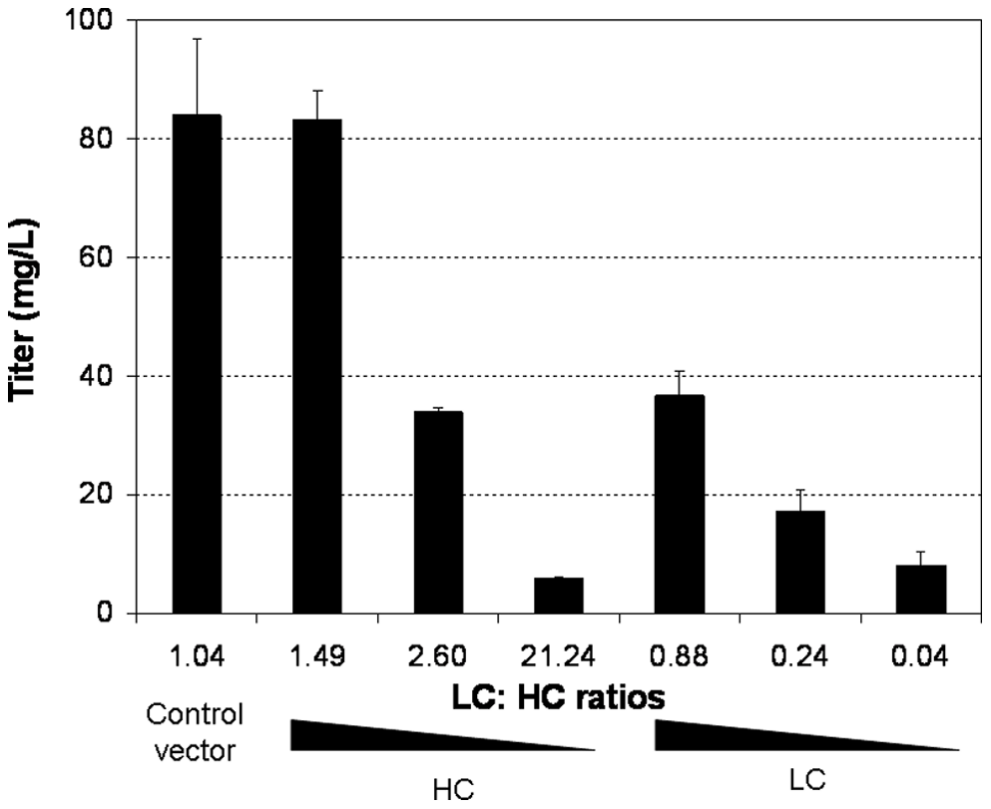
Effect of LC:HC ratios on mAb productivity in stable transfection pools. CHO DG44 stable transfection pools at different LC:HC ratios generated were cultured in shake flask batch cultures. Titers of monoclonal antibody at the end of culture were determined using a nephelometric method or ELISA. Each value represents the average and standard deviation of measurements from two independent stable transfection pools.

The impact of LC:HC ratio on mAb yield was analyzed by plotting the end-point titer in shake flask batch culture against the LC:HC ratios as determined by ELISA (Figure 5). The control vector containing the IRESwt which controlled LC:HC ratio at 1.04 gave the highest titer of 84.1 mg/L. Slightly increasing the ratio to 1.49 by decreasing HC expression did not change titer significantly, yielding 83.3 mg/L. Further increasing the LC:HC ratios to 2.60 and 21.24 by decreasing the HC expression dramatically reduced the mAb titers to 34.0 and 5.9 mg/L, respectively. In contrast, a slight decrease of LC:HC ratio to 0.88 by decreasing the LC expression resulted in a sharp decline of mAb titer to 36.8 mg/L. The titer dropped to 17.1 and 8.1 mg/L when LC:HC ratios decreased to 0.24 and 0.04 by further reducing LC expression.

**Figure 6:**
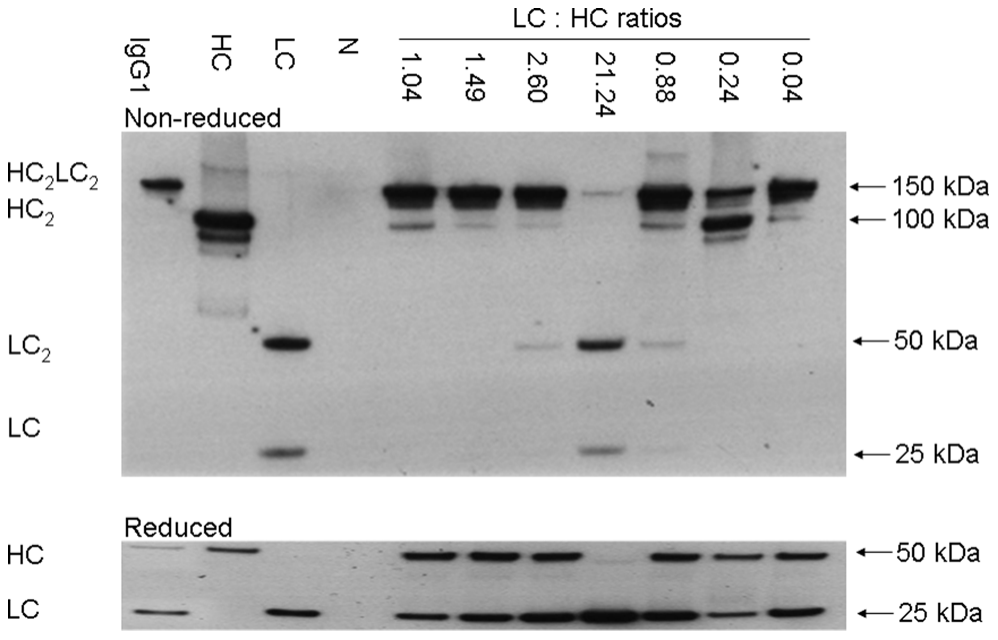
Western blot analysis of supernatant in stable transfection pools at different LC:HC ratios. CHO DG44 stable transfection pools at different LC:HC ratios were cultured in shake flask batch cultures. Crude supernatant collected at the end of culture was analyzed under both non-reducing and reducing conditions by western blot. Positive and negative controls are the same as described in Figure 4.

The qualities of mAb produced in stable pools at different LC:HC ratios were first characterized by western blotting analysis of culture supernatant under non-reducing conditions (Figure 6). Supernatant containing equal amounts of mAb determined by ELISA was loaded into each lane of a NuPAGE gel. Lesser amount of mAb was loaded for sample at the LC:HC ratio of 21.24 to avoid overexposure of LC fragments. Product from the control vector with the LC:HC ratio of 1.04 contained complete IgG monomers HC_2_LC_2_ and a slight band corresponding to HC_2_ dimers, suggesting inefficient usage of HC polypeptides in mAb synthesis. Increasing LC:HC ratios to 1.49 and 2.60 by reducing HC expression resulted in secretion of less HC_2_ dimers. Further increasing the ratio to 21.24 eliminated secretion of HC_2_ dimers but resulted in secretion of LC_2_ dimers and LC monomers besides the complete IgG monomers, suggesting inefficient usage of LC polypeptides in mAb synthesis. In contrast, decreasing LC:HC ratios to 0.88, 0.24, and 0.04 resulted in secretion of HC_2_ dimers together with the complete IgG monomer, suggesting inefficient usage of HC polypeptides in mAb synthesis. Western blotting analysis of reduced products in supernatant was next performed. LC and HC polypeptides expressed from all different IRES variants had similar sizes compared to standard LC and HC polypeptides. HC band at the LC:HC ratio of 21.24 was not observed due to lower amount of sample loading.

To obtain a more accurate protein characterization, protein A purified mAb in stable pools at different LC:HC ratios were separated on SDS-PAGE under reducing conditions. The excised bands corresponding to LC and HC polypeptides were digested by trypsin and then analyzed on LC-MS/MS for determining the signal peptide cleavage sites. The N-terminal tryptic peptide sequences of both LC and HC were confirmed by MS/MS via *de novo* and database matching using PEAKS program. Detection of peptide DIQMTQSPSSLSASVGDR and EVQLVESGGGLVQPGGSLR indicated that signal peptides of LC and HC were both cleaved at correct sites. No miscleaved signal peptide was observed on LC and HC in stable pools at LC:HC ratios of 1.04, 1.49, 2.60, and 0.24. LC-MS/MS analyses of products in stable pools at LC:HC ratios of 21.24 and 0.04 were not performed because of insufficient product.

**Figure 7:**
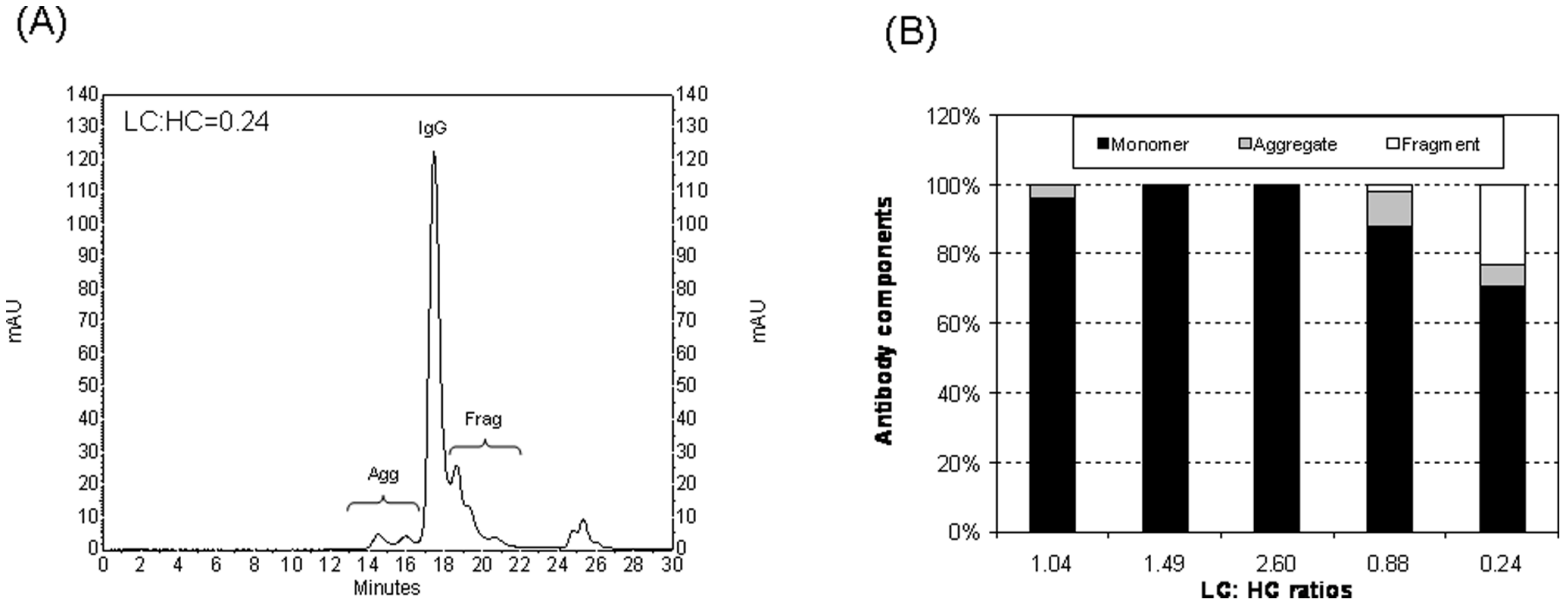
Representative size exclusion chromatogram and distribution of complete IgG monomer, aggregate, and incomplete IgG fragments produced in stable transfection pools at different LC:HC ratios. Components within protein A purified supernatant collected at the end of culture were separated by size exclusion chromatography followed by the identification and quantification of species by light scattering and UV detection, respectively. Analysis was done for duplicate stable transfection pools. (A) Representative chromatogram obtained by UV detector for the pool of LC:HC = 0.24. Agg: Aggregates; IgG: complete IgG monomer; Frag: Incomplete IgG fragments. (B) Quantitative comparison of complete IgG monomer, aggregates, and incomplete IgG fragments for different LC:HC ratios. Each value in figure B represents the average of four measurements from two independent stable transfection pools.

Finally, the impact of LC:HC ratio on mAb aggregation was analyzed by using SEC coupled to a dynamic light scattering detector and UV detector. Supernatant collected at the end of culture was purified by protein A affinity chromatography before SEC analysis. Analysis of products in stable pools at LC:HC ratios of 21.24 and 0.04 were not performed due to insufficient product. One representative UV chromatogram for the LC:HC ratio of 0.24 was shown in Figure 7A. The molecular weight of each peak was calculated based on hydrodynamic radius determined by light scattering (data not shown). Peaks with average molecular weight greater and lower than the complete IgG monomers were grouped as aggregates and incomplete mAb fragments, respectively. Relative mass amounts of aggregate, complete IgG monomer, and incomplete mAb fragment were quantified using the respective peak area under the UV chromatograms. Analysis was done for duplicate stable transfection pools at each LC:HC ratio. Average distributions of components at different LC:HC ratios were shown in Figure 7B. Product at LC:HC ratio of 1.04 contained 96.2% IgG monomers, 3.6% aggregates, and 0.2% fragments. Increasing the ratio to 1.49 and 2.60 resulted in increase of IgG monomers to more than 99.3% and decrease of both aggregates and fragments to less than 0.5%. In contrast, decreasing the LC:HC ratio to 0.88 resulted in decrease of IgG monomers to 88.1% and increase of aggregates to 10.1% and fragments to 1.8%, respectively. Further decreasing the ratio to 0.24 did not worsen aggregation but saw a sharp increase of fragments to 23.3% and decreasing IgG monomers to only 70.6%.

## Discussion

Translation represents a critical step in regulating gene expression in mammalian cells. During cap-dependent translation, ribosomes can recognize non-AUG triplets as start codons and initiate translation with reduced efficiency. The magnitude of reduction varies depending on the type of non-AUG triplets and their sequence context [34], [35], [36]. EMCV mRNA translation is initiated at AUG-11 or AUG-12 and less frequently at AUG-10 on internal ribosome entry sites (IRES) by a cap-independent mechanism [32]. We hypothesized that non-AUG initiated translation efficiency would be affected in similar ways in both cap-dependent and cap-independent translation. Mutation of AUG to non-AUG codons would reduce IRES translation efficiency and provide for greater precision during control of gene expression. Consistent with reports in cap-dependent translation studies, IRES variants with different non-AUG codons exhibited different strengths. However, the order of translation efficiency exhibited by different non-AUG was not the same as that reported in a previous study (Table II) [35]. Although we observed that mutating AUG-11 to CUG resulted in the lowest drop in translation efficiency, IRES variants with GUG-11, ACG-11, AUA-11, and UUG-11 did not exhibited strength differences as significant as those reported. One possible explanation is that when AUG-11 was mutated, efficient translation could be initiated at AUG-10 and AUG-12 [32]. When either both AUG-11 and AUG-12 or all three AUGs were mutated, the order of non-AUG codons for translation efficiency is the same as that in cap-dependent translation except for GUG. Conflicting orders for non-AUG initiation efficiency had been reported in different cap-dependent translation studies [34], [35], [36]. The different order of efficiency could be due to differences in sequence context around the start codons.

**Table II:** Comparison of non-AUG translation efficiency in cap-dependent and cap-independent translations.

Start condon	Cap-dependent translation efficiency (%)^a^	EMCV IRES-mediated cap-independent translation efficiency (%)		
		Mutation of 11^th^ AUG	Mutation of 11^th^ and12^th^ AUG	Mutation of 10^th^, 11^th^ and 12^th^ AUG
AUG	100	100.00	100.00	100.00
CUG	50	45.18	9.47	13.58
GUG	20	35.48	0.98	1.37
ACG	10	33.25	4.02	3.23
AUA	5	39.91	1.79	1.67
UUG	ND	34.39	0.58	0.81

a Cap-dependent translation efficiencies of non-AUG condons were determined in COS-1 cells in transient transfections [35].

It has been reported that EMCV IRES variants generated by mutations of the critical domains in IRESwt exhibited cell-specificity [28]. Four IRES mutants that exhibited up to 36% difference in strength in HEK293T cells showed no apparent difference in CHO K1 cells. One possible explanation is that the strengths of these IRES variants depend on the interaction between *trans-acting* protein factors, such as polypyrimidine tract binding protein and the critical protein binding domains in EMCV IRES [42]. The expression level of these protein factors in CHO K1 cells may be lower than in HEK293T cells, resulting in decreased sensitivity of IRES strength to mutations of the critical domains. In contrast, the strengths of our newly generated EMCV IRES variants were manipulated by mutation of translation initiation sites, which may result in changes in ribosome binding affinity to the translation initiation start codon. As the molecular mechanism of translation initiation is highly conserved in all mammalian cells, it is not surprising that our IRES variants had similar relative strength in different cell lines.

We successfully controlled the LC over HC expression across a wide range of values using our IRES variants in stable transfections (Figure 4). Independent alteration of LC and HC expression provided better understanding of the mAb synthesis kinetics. Consistent with previous reports that expression of extra LC is more efficient for mAb expression [7], [43], [44], we found that at a balanced LC:HC ratio of 1.04, the mAb synthesis was not efficient as indicated by the secretion of HC fragment and occurrence of aggregates although this ratio gave the highest mAb titer (Figure 5). mAb production levels did not drop with a slight reduction of HC polypeptides, increasing LC:HC ratio from 1.04 to 1.49, but both aggregation and secretion of incomplete mAb fragment were minimized (Figure 6 and 7). In contrast, reduction of LC polypeptides by a similar magnitude (from 1.04 to 0.88) resulted in a sharp decrease in mAb production, a dramatic increase in aggregate, and secretion of HC fragments. It is known that mAb folding and assembly is more efficient when having extra LC polypeptides [44]. A slight reduction of HC polypeptides reduced the availability of substrate for mAb synthesis but having extra LC polypeptides enhances mAb folding and assembly, as a result mAb production level was not changed. However, the enhanced mAb folding and assembly reduced aggregation and enhanced the efficiency of LC and HC polypeptides usage for mAb synthesis. Similar mechanisms where a balance between substrate availability and folding efficiency is needed may explain the sharp decline in mAb production when LC polypeptides were slightly reduced, which not only reduced the substrate availability but having extra HC polypeptides may also reduce mAb folding and assembly, thus resulting in a sharp decrease in mAb titer and increase in aggregate. Substantial decrease of LC or HC polypeptides both resulted in sharp drop of mAb titers, as the amounts of substrates for mAb synthesis have become limiting. Also, extreme deviations from the balanced LC:HC ratios resulted in inefficient use of either LC or HC polypeptides for mAb synthesis. As such, we propose an optimized vector for mAb expression should express both LC and HC genes at high level and control the LC expression in slight excess.
